# Sclerokeratoplasty as the Therapy for Corneal Perforation due to Exposure and Neurotrophic Keratopathy

**DOI:** 10.1155/2014/467249

**Published:** 2014-01-15

**Authors:** Radoslaw Rozycki, Izabela Nowak-Gospodarowicz, Dominika Bialas, Rafal Pawlik, Marek Rekas

**Affiliations:** Ophthalmology Department, Military Institute of Medicine, 128 Szaserow Street, 04-141 Warsaw, Poland

## Abstract

A case report of exposure and neurotrophic keratopathy after acoustic neuroma surgery resulting in perforation if not managed appropriately and timely is presented. Sclerokeratoplasty on 360 degrees may be an effective treatment method of corneal perforation in complete anaesthetic cornea when the standard penetrating keratoplasty failed. At a 12-month follow-up, the patient is doing well. UCVA is 0.5, the IOP is normal, and the graft remains clear. Systemic immunosuppression is the main disadvantage of this method. Further investigation is needed to assess the effectiveness and safety of this method.

## 1. Introduction

Acoustic neuromas (Vestibular Schwannomas) (VS) are oncologically benign tumours which constitute more than 90% of all cerebellopontine angle tumours and more than 10% of all primary brain tumours. Surgical excision of these tumours is one of the most challenging neurosurgical procedures because of their location close to vital structures such as the anterior inferior cerebellar artery (AICA) or the 7th and 8th cranial nerves [1]. When the tumour exceeds 3 cm, it might involve the trigeminal nerve causing a depressed corneal reflex, which is accompanied by peripheral facial nerve paresis leading to the development of exposure and neurotrophic keratopathy. This condition, especially with poor Bell's phenomenon, is usually resistant to conventional therapies and has a very unfavourable prognosis. Loss of the sensory innervation of the cornea decreased the number of corneal stem cells [2], decreased metabolic and mitotic rates in the corneal epithelium, and reduced acetylcholine and choline acetyltransferase concentrations [3, 4] resulting in the development of persistent epitheliopathy.

This chronic epithelial breakdown enables proteolytic enzymes to degrade the extracellular matrix components because they cannot protect corneal structural and signaling matrix proteins anymore. This condition may progress to corneal ulceration, perforation, and loss of the eye.

The ophthalmic goal of treatment is to protect the cornea from external irritating factors, to stop its progressive degradation, and to support its healing.

## 2. Case Report

The patient was a 64-year-old female with a 4-year history of exposure and neurotrophic keratopathy in the right eye due to unresolved peripheral facial nerve and trigeminal nerve palsies after acoustic neuroma surgery. The patient underwent bilateral cataract surgery at the age of 61 and, except for mild hypertension, remained healthy. After 2 years of satisfactory treatment of lagophthalmos with a gold eyelid weight, it was necessary to remove the weight from the right upper eyelid in order to perform an MRI scan. Despite the use of moisturizing drugs and eye taping, severe corneal ulcer developed 6 months after the removal of the weight. After 2 months of ineffective conservative treatment, the patient was referred to our clinic.

On admission, the corrected distance visual acuity (CDVA) was 0,01 (Snellen chart) and intraocular pressure (IOP) was 14 mmHg in the right eye. CDVA was 1,0 in the left eye.

**Figure 1:**
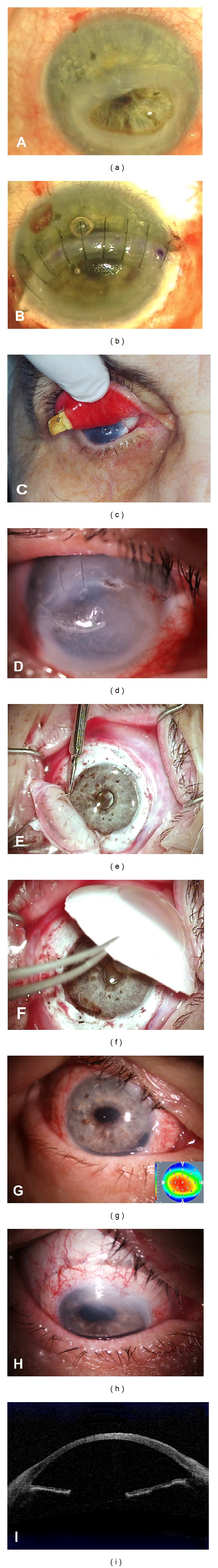
The anterior segment of the eye 12 months after sclerokeratoplasty on 360 degrees: pre- and postoperative picture and the surgical technique. (a) Corneal perforation (b) inferiorly decentred penetrating keratoplasty; (c) extrusion of the gold weight from the right upper eyelid; (d) graft rejection; (e) sclerokeratectomy (preparation of the cornea); (f) sclerokeratectomy (graft adaptation); (g) anterior segment of the eye and pachymetry map 12 months after surgery; (h) donor graft fitting 12 months after surgery; (i) anterior segment of the eye 12 months after the surgery (OCT-Visante).

Peripheral right facial nerve palsy, lagophthalmos of 5 millimetres with paralytic ectropion, poor Bell's phenomenon, and complete corneal anaesthesia were noted in the right eye. Slit lamp examination revealed ulceration with descemetocele in the lower part of the cornea in the right eye. The intraocular lens (IOL) was in place and other ocular structures and the left eye were without any pathological changes. Urgent amniotic membrane transplant (AMT) and complete tarsorrhaphy were performed in the right eye. The patient was discharged home on topical 0,5% levofloxacin and was followed-up in an outpatient clinic. 15 days after initial clinical improvement and partial removal of the tarsorrhaphy sutures the patient came to our emergency room with a corneal perforation in the right eye (Figure 1(a)). The patient noticed vision deterioration and admitted to having touched her cornea during the instillation of the eye drops. The patient was admitted to our clinic. Urgent inferiorly decentred penetrating keratoplasty was performed ([5], Figure 1(b)) due to the size of the perforation, its localisation, and vascularisation of the lower limbus. A new 1,8 g of gold weight (safe for MRI) was placed in the pretarsal space of the right eye and a correction of the paralytic ectropion was performed. Triple systemic immunosuppression (cyclosporine A, mycophenolate mofetil, and prednisone) was administered. After 1 month, the gold weight was extruded from the scarred tissue of the right upper eyelid (Figure 1(c)). Despite eye taping, systemic immunosuppression, and visually normal upper limbus, no reepithelialization was noted. Graft rejection with scleral melt in the suture localisation occurred 3 months after the surgery (Figure 1(d)). Urgent sclerokeratoplasty on 360 degrees was performed in the right eye. Under general anesthesia, the recipient sclera was incised circumferentially 4 mm distally from the limbus and to 2/3 of its thickness in depth. The anterior chamber was opened and filled with viscoelastic material. Anterior synechiae were lysed and the recipient cornea was removed with curved scissors 1 mm peripheral from the limbus. The adequate donor sclerocorneal graft was sutured to the recipient sclera with 10.0 nylon sutures. The anterior chamber was reformed with viscoelastic. Special care was taken to avoid injury of structures of the angle. The conjunctiva was reattached to the donor limbus ([6–9], Figures 1(e) and 1(f)).

The immunosuppression scheme was introduced by the specialist in organ transplantations from our institute and included orally: cyclosporine A (CsA) 100 mg bid varying to 50 mg bid depending on drug concentration level in blood, mycophenolate mofetil (MMF) 1000 bid, and prednisone 40 mg daily for 1 month and then tapered to 8 mg per day until completion in POM12; locally: prednisolone acetate 1% 1 gtt q2h daily limited to 1 gtt qid from POM3.

At a 12-month follow-up, the patient is doing well (Figures 1(g), 1(h), and 1(i)). CDVA in the right eye is 0.5 (Snellen chart) and intraocular pressure (IOP) is 16 mmHg. The graft remains clear. No complications of the systemic immunosuppression have been noted. The patient receives regular follow-up in our clinic.

## 3. Discussion

This case shows how complicated the treatment of severe exposure and neurotrophic keratopathy after excision of the cerebellopontine angle tumour might be.

To our best knowledge, there are no standards for treatment of this condition.

Restoration of complete eyelid closure is crucial in order to prevent the cornea from mechanical injuries and enable proper impact of therapeutic agents.

Depending on the severity of impairment of the ocular surface, vitamins, collagenase inhibitors, anti-inflammatories and tear substitutes, cyclosporine, autologous serum, or grafts of the amniotic membrane may be used. Recent studies have also shown promising results on the use of nerve growth factor (NGF) [10–12], matrix proteins, and bioengineered matrix regenerating agent (RGTA) [13].

In this case, initial AMT with tarsorrhaphy brought about clinical improvement and therefore corneal perforation came as a surprise to us. The patient admitted that she might have touched the cornea during instillation of the eye drops. We believe that this is the direct cause of the perforation.

Classic treatment of corneal perforation involves the use of glue, the smallest patch grafts, and tectonic grafts [14, 15]. After the first urgent decentred penetrating keratoplasty, the visual outcome was disappointing (the finger counting level). Despite systemic triple immunosuppression, there was no reepithelialization of the cornea and the graft was rejected. Moreover, apart from vascularization of the lower part of the corneal graft and limbus, we observed scleral melt on the graft recipient border. These complications propelled us to look for a technique which would enable harvesting a graft big enough to cover all the impairments which were to be removed, might give better visual outcomes due to positioning the sutures not in the visual axis, and would graft limbal stem cells. That is why we decided to perform sclerokeratoplasty on 360 degrees. We believe that the final effect of this procedure may corroborate the rationale for our treatment paradigm.

Systemic immunosuppression is the main disadvantage of this method. There are no consistent details concerning immunosuppressive treatment, dosage, and its duration. From many different schemes, we concluded that at least two systemic immunosuppressive agents are necessary to prevent sclerocorneal graft from rejection [16–21]. In our case, we followed the orders of the specialist in organ transplantations from our institute based on recommendations of the European Society of Organ Transplantation (ESOT) [22].

Although sclerokeratoplasty is not a standard procedure for the treatment of corneal perforation, it may be effective in some cases of the perforation of an anaesthetic cornea, especially when the standard penetrating keratoplasty is of poor prognosis or impossible to be performed due to the anatomy of the lesions. Further investigation is needed to confirm the effectiveness and safety of this method.

This paper was accepted as a scientific poster for the European Society of Ophthalmology Congress (SOE), Copenhagen, Denmark, 8–11 June 2013, and was presented at the 5th International Symposium “Advances in diagnosis and treatment of corneal diseases,” Wisla, Poland, 7–9 March 2013.
